# Larval Feeding Habits of Five Firefly Species Across Aquatic, Semi-Aquatic, and Terrestrial Lineages

**DOI:** 10.3390/insects15121004

**Published:** 2024-12-18

**Authors:** Lin-Yu Yang, Da-Rui Tang, Fu-Xin Li, Shi-Qi Luo, Cheng-Quan Cao, Qi-Lin Zhang

**Affiliations:** 1 Faculty of Life Science and Technology, Kunming University of Science and Technology, Kunming 650500, China; yanglinyu1214@163.com (L.-Y.Y.); 18314112564@139.com (D.-R.T.); m18819776113@163.com (F.-X.L.); lsqsc7777777@163.com (S.-Q.L.); 2 College of Life Sciences, Leshan Normal University, Leshan 614004, China

**Keywords:** fireflies, feeding habits, feeding range, feeding preference, habitat types

## Abstract

While adult fireflies are terrestrial, their larvae inhabit various habitat types, and a lack of comprehensive research on the feeding habits of these larvae across different habitats has greatly impeded the development of artificial diets. Here, we tested 14 types of foods, primarily covering gastropods, vertebrates, and fruit, to survey feed for aquatic (*Aquatica leii* and *Sclerotia substriata*), semi-aquatic (*Pygoluciola qingyu* and *Pygoluciola* sp.), and terrestrial (*Pyrocoelia analis*) fireflies. The results show that *A. leii*, *S. substriata*, *P. qingyu*, *Pygoluciola* sp., and *P. analis* fed on 12, 6, 10, 10, and 7 different foods, respectively, showing an obvious difference in feeding range among various inhabit types of fireflies. Aquatic lineages preferred to consume freshwater snails, followed by pork meat and land slugs, while semi-aquatic fireflies favored freshwater snails, followed by fish and shrimp. Favorite foods were thus freshwater snails such as *Cipangopaludina chinensis* for both aquatic and semi-aquatic fireflies, but feeding preference differed for second favorite foods (e.g., pork vs. fish meat). Terrestrial *Pyrocoelia analis* showed different feeding preferences compared withthe other two habitat lineages, with terrestrial snails and slugs as their favorite foods, followed by freshwater snails, such as *C. chinensis*. These findings not only uncovered larval feeding habits of fireflies across various inhabit types but also indicated that readily available and affordable *C. chinensis* products can serve as wild snail alternatives in the artificial feeding of fireflies. This study is the first to explore the feeding habits of firefly species inhabiting water and land habitat types, adding to the understanding of the feeding characteristics of fireflies.

## Simple Summary

Clarification of the feeding habits (i.e., feeding range and preference) of larval fireflies inhabiting various habitats (aquatic, semi-aquatic, and terrestrial) is key for the development of artificial diets and the indoor rearing of fireflies. The two aquatic species, *Aquatica leii* and *Sclerotia substriata*, prefer to prey on freshwater snails, but their feeding range presents obvious differences. The two semi-aquatic fireflies, *Pygoluciola qingyu* and *Pygoluciola* sp., show similar feeding habits, preferring freshwater snails, followed by freshwater fish meat, while the terrestrial firefly *Pyrocoelia analis* prefers land slugs, followed by freshwater snails. Overall, the feeding habits of larval fireflies were distinct, altering with habitat type. The common and easily accessible freshwater snail *Cipangopaludina chinensis* is a potential food for aquatic, semi-aquatic, and terrestrial fireflies in indoor artificial rearing, possibly circumventing the current shortage of wild-snail-derived foods.

## 1. Introduction

‘Fireflies’ refers to all the members of the Lampyridae family (Coleoptera), representing more than 2000 species and 100 genera [1,2,3]. They are divided into aquatic, semi-aquatic, and terrestrial lineages according to their larval habitats [4]. Only the larvae of aquatic species, such as *Aquatic leii* and *Sclerotia substriata*, show morphological adaptations to freshwater (e.g., branched tracheal gills and smooth and soft bodies [5,6]). These species also show transcriptomic and metabolomic adaptations to aquatic life [3,7] compared with the terrestrial and semi-aquatic lineages. Using four species of freshwater snails (*Gyraulus convexiusculus*, *Lymnaea stagnalis*, *Oncomelania hupensis*, and *Bellamya purificata*), crucian carp fish (*Carassius auratus*), and pork meat as candidate foods, previous studies found that *A. leii* preferred to feed on *O. hupensis* [8,9]. *Pygoluciola* is recognized as a semi-aquatic firefly genus, and its feeding habits have not been studied. However, the feeding habits of *P. qingyu* were inferred as dead freshwater snails and small invertebrates through preliminary field observations by us in Ebian County, Sichuan Province, China, in 2022. Some terrestrial species have been employed as potential biological control against terrestrial snails, for example, the control of *Bradybaena ravida* by the genus *Pyrocoelia* (e.g., *Pyrocoelia analis* and *Pyrocoelia pectoralis*) [10,11]. Both in nature and in the laboratory, larval *P. pectoralis* seem to primarily feed on two species of living and fresh land snails, *Bradybaena similaris* and *B. ravida* [12]. Thirteen species of gastropods have been reported as prey of larval *Alecton discoidalis*, a species of terrestrial firefly endemic to Cuba [13].

Collectively, the larval feeding habits of fireflies have been surveyed intermittently over the past two decades, with a focus on one–two species from the same habitat type, the description of their biological characteristics, and the development of potential biological agents to control pest gastropods. This has impeded the development of feeds available for indoor rearing due to an actual lack of natural gastropod-derived foods and the restricted availability of candidate foods. Therefore, additional studies covering more types of candidate foods, more firefly species, and more habitat types are essential for a better understanding of the feeding habits of firefly lineages.

Importantly, in recent years, wild firefly populations have declined dramatically due to habitat destruction, artificial light, and commercial capture [14,15]. Raising fireflies indoors and later releasing them can be one of the conservation strategies, and the development of artificial diets can promote the indoor rearing of fireflies. However, the problems with using wild snails as feed are firstly that large numbers of snails are difficult to obtain in the wild due to variability in population size with environmental conditions (e.g., season, geographic location, and human interference). Secondly, the large-scale indoor feeding of wild snails remains a challenging task, and the cost therein is high due to their unpopular demand and market. Thus, a survey of larval feeding habits will determine if there are more accessible and economical alternative foods to wild snails that are in more stable supply for use in artificial feeding.

In this study, we experimentally surveyed the larval feeding habits (particularly feeding range and preference) of five firefly species inhabiting aquatic (*A. leii* and *S. substriata*), semi-aquatic (*P. qingyu* and *Pygoluciola* sp.), and terrestrial environments (*P. analis*) in the laboratory using 14 different foods (e.g., gastropods and insects, common vertebrate and snail meat, and fruits). The results provide valuable information for the understanding of the feeding habits of firefly larvae and the improvement of indoor rearing technology for their conservation.

## 2. Materials and Methods

### 2.1. Fireflies and Foods

Larvae of *A. leii*, *S. substriata*, *P. qingyu*, *Pygoluciola* sp., and *P. analis* were obtained from the Culture Centre of Fireflies, Ganzhou, Jiangxi Province, China. These fireflies were kept in the Faculty of Life Science and Technology, Kunming University of Science and Technology, Kunming, China. Here, 4th instar fireflies were used, as these can feed and move optimally.

In total, seven fresh foods were prepared for the food preference testing experiment using all five species of fireflies, following previous studies [8,9,10,16]. These foods covered gastropods, including wild freshwater snails (*Sinotaia quadrata* and *Margarya melanioides*), wild terrestrial snails (*B. ravida* and *B. similaris*), and a wild slug (*Agriolimax agrestis*), as well as ordinary meats such as pork (*Sus*) muscle and freshwater fish (*C. auratus*) muscle. The pork and fish meats have higher concentrations of amino acids than snails [9], showing their potential as nutritional additives in the indoor rearing of fireflies; thus, they were selected as the tested food samples in this study.

Moreover, seven additional types of foods were added to this study, including widely cultivated and popularly commercialized freshwater snails (*Cipangopaludina chinensis*) in China, insects (*Aedes aegypti*, *Pheidole megacephala*, and *Tenebrio molitor*), freshwater shrimp (*Macrobranchium nipponense*) muscle, the common and readily available earthworm (*Eisenia foetida*) from markets, and fruits (coconut *Cocos nucifera*). These raw materials were dissected and separated before use to remove inedible components. Detailed information (e.g., market price, food status, and reason for choosing these foods) of all the foods tested in this study is presented in Supplementary Table S1.

### 2.2. Food Preference Testing Experiment

The experimental design followed a previous study with minor modifications [17]. In total, the 14 candidate foods were placed in the bottom of a box (44 cm × 33 cm × 16.7 cm) in a circle. After fasting for 24 h, the fireflies were released in the center of the circle to choose their preferred food (Supplementary Figure S1). The initial weight of each food was 6 g, and the experiment was repeated three times independently as three biological replicates for each species, with 100 individuals per replicate. In the food preference testing experiment for aquatic firefly lineages, the foods were placed into small uncovered micropore (size of 200 mesh) containers (circular, stainless steel, diameter at 2 cm and depth at 1.5 cm) to avoid food diffusion in water, with the depth of water at 2 cm. In addition, in the food preference testing experiment for semi-aquatic firefly lineages, water was sprayed approximately every 20 min to maintain the moisture in the experimental environments and to avoid obvious water accumulation.

The food preference testing experiments were conducted at 25 °C, 75% humidity, and an L:D = 14 h:10 h photoperiod, following previous publications [8,10,16]. Food spoilage was detected after 24 h, including the rapid rotting of fish and pork meat. The experiment was thus terminated at this time, and the remaining foods were collected separately. In the test for terrestrial lineages, in order to eliminate water evaporation of foods, we slightly sprayed water mist on the surface of foods (once approximately every 1 h for earthworms and muscle of freshwater animals, and once every 2 h for the other foods). The amount of food consumed was calculated with the following formula: food initial weight (6 g)—weight of the remaining foods [18,19]. Notably, unlike in terrestrial environments, the weight of the foods increases as a result of water uptake in aquatic and semi-aquatic environments. Therefore, the weight of water spontaneously absorbed in foods was considered and subtracted for the food consumption calculations of aquatic and semi-aquatic lineages. The weight of each type of food consumed by aquatic and semi-aquatic fireflies was thus calculated as follows: initial weight + weight of the absorbed water for 24 h—weight of the remaining foods. The weight of the absorbed water over 24 h for each type of food in the aquatic and semi-aquatic environments was different and was determined as follows: the variance value of food weight before and after exposure to water (detailed values are presented in Supplementary Table S2).

### 2.3. Statistical Analysis

The results are presented as the mean of three biological replicates ± standard error (S.E). In addition, the food with the highest consumption amount serves as a control in recipes of fireflies; thus, the data were presented as a percentage of the controls. Multiple comparisons were performed using a One-Way Analysis of Variance (ANOVA), followed by Duncan’s test in Prism 8.0.2 (GraphPad Software Inc., La Jolla, CA, USA). Differences between groups were considered to be significant at a *p*-value < 0.05.

## 3. Results

**Figure 1:**
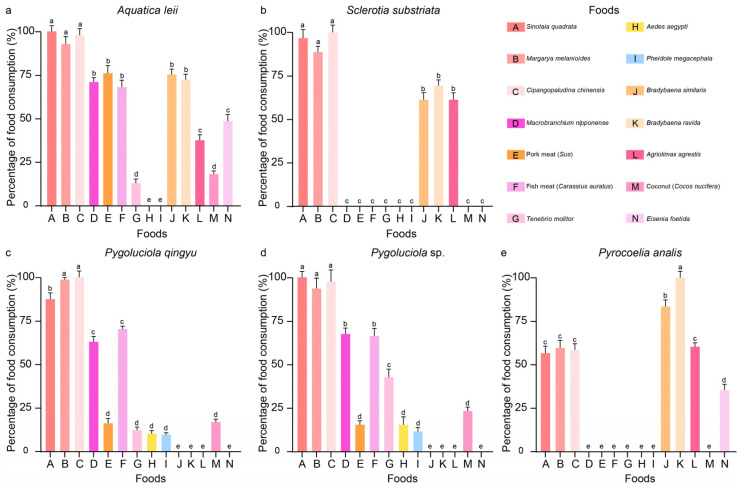
The percentage of food consumption of the 100 4th instar larvae of *Aquatica leii* (**a**), *Sclerotia substriata* (**b**), *Pygoluciola qingyu* (**c**), *Pygoluciola* sp. (**d**), and *Pyrocoelia analis* (**e**) against *Sinotaia quadrata*, *Margarya melanioides*, *Cipangopaludina chinensis*, *Bradybaena ravida*, *Bradybaena similaris*, *Agriolimax agrestis*, *Macrobranchium nipponense*, *Aedes aegypti*, *Pheidole megacephala*, *Tenebrio molitor*, pork (Sus) meat, fish (*Carassius auratus*) meat, earthworm (*Eisenia foetida*), and coconut (*Cocos nucifera*) flesh in 24 h. The food with the highest consumption amount in the recipes of each species of fireflies serves as a control (100%). Error bars represent S.E. of three biological replications. Different letters above the data columns indicate significant differences at the 0.05 level.

The feeding range of the aquatic *A. leii* larvae included 12 types of foods (Figure 1a), namely three species of freshwater snails, two species of terrestrial snails, *T. molitor*, slugs, pork and crucian carp meat, shrimp, earthworms, and coconut. The aquatic *S. substriata* only consumed six types of foods, including three species of freshwater snails, two species of terrestrial snails, and slugs (Figure 1b). The same feeding range was detected between *P. qingyu* (Figure 1c) and *Pygoluciola* sp. (Figure 1d), consuming 10 types of foods, including three species of freshwater snails, three species of insects, shrimp, coconut, pork, and crucian carp meat. Meanwhile, seven types of foods were consumed by larval *P. analis* (Figure 1e), including three species of freshwater snails, two species of terrestrial snails, slugs, and earthworms.

For aquatic fireflies, *A. leii* showed the highest consumption amount for three species of freshwater snail (feeding consumption reached 4.60 ± 0.15 g, 100%, 4.50 ± 0.17 g, mean percent 97.83%, and 4.28 ± 0.19 g, 93.04% for *S. quadrata*, *C. chinensis*, and *M. melanioides*, respectively), without significant (*p* > 0.05) consumption differences between them (Figure 1a and Supplementary Figure S2a), followed by pork (3.50 ± 0.20 g, 76.09%) and crucian carp (3.12 ± 0.17 g, 71.09%) meat, two species of terrestrial snails (3.47 ± 0.12 g, 75.43% and 3.32 ± 0.18 g, 72.17% for *B. similaris* and *B. ravida*, respectively), and shrimp (3.27 ± 0.11 g, 71.09%). The difference between the freshwater snail and pork, crucian carp, terrestrial snails, and shrimp meat was significant (*p* < 0.05). The lowest feeding foods for *A. leii* were three species of insects and coconut (0.82 ± 0.09 g, 17.61%), with the larvae not eating any *A. aegypti* or *P. megacephala*. The highest food consumption of *S. substriata* was three species of freshwater snails (2.06 ± 0.07 g, 100%, 2.00 ± 0.10 g, 97.09%, and 1.84 ± 0.06 g, 89.32% for *S. quadrata*, *C. chinensis*, and *M. melanioides*, respectively) (Figure 1b and Supplementary Figure S2b), with significantly (*p* < 0.05) more of these being consumed compared with other foods, followed by two species of terrestrial snails (1.44 ± 0.06 g, 69.90% and 1.26 ± 0.09 g, 61.17% for *B. ravida* and *B. similaris*, respectively) and the slug *A. agrestis* (1.25 ± 0.08 g, 60.68%). Compared with *A. leii*, larval *S. substriata* did not feed on any of the foods, such as pork and crucian carp meat, shrimp, or coconut.

For the semi-aquatic fireflies, *P. qingyu* showed the highest feeding amount for two species of freshwater snails belonging to the Viviparidae family, including *C. chinensis* (5.07 ± 0.15 g, 100%) and *M. melanioides* (5.00 ± 0.05 g, 98.62%), followed by another pond snail *Sinotaia quadrata* (4.42 ± 0.18 g, 87.18%) (significance between consumption amounts, *p* < 0.05) (Figure 1c and Supplementary Figure S2c). *Pygoluciola* sp. larvae also showed the highest feeding amount for these three species of freshwater snails (*p* > 0.05) (2.57 ± 0.07 g, 100%, 2.50 ± 0.18 g, 97.28%, and 2.40 ± 0.15 g, 93.38% for *S. quadrata*, *C. chinensis*, and *M. melanioides*, respectively) (Figure 1d and Supplementary Figure S2d). Food consumption of *P. qingyu* for crucian carp fish and freshwater shrimp is 3.57 ± 0.06 g, 70.41% and 3.19 ± 0.15 g, 62.91%, respectively, and that of *Pygoluciola* sp. for these two types of meats is 1.70 ± 0.12 g, 66.15% and 1.73 ± 0.08 g, 67.32%, followed by three species of insects (feeding consumption of *P. qingyu* for *T. molitor*, *A. aegypti*, and *P. megacephala* was 0.61 ± 0.09 g, 12.03%, 0.52 ± 0.08 g, 10.26%, and 0.50 ± 0.06 g, 9.86%, respectively; *Pygoluciola* sp. consumption was 1.09 ± 0.11 g, 42.41%, 0.40 ± 0.12 g, 15.56%, and 0.31 ± 0.06 g, 12.06%), while pork consumption was only 0.81 ± 0.18, 15.98% and 0.4 ± 0.09, 15.56% (*p* < 0.05), respectively, without any consumption of terrestrial snails, the slugs, or the earthworms.

For the terrestrial lineages, *P. analis* larvae presented the highest feeding capacity for *B. ravida* (5.63 ± 0.20 g, 100%), followed by another land snail belonging to the same genus, *B. similaris* (4.70 ± 0.21 g, 83.48%), with a significant (*p* < 0.05) difference in the consumption levels between these two terrestrial snails (Figure 1e and Supplementary Figure S2e). The second consumption food of *P. analis* was the slug *A. agrestis* (3.40 ± 0.12 g, 60.39%) and three species of freshwater snails (*M. melanioides*, *C. chinensis*, and *S. quadrata* with consumption values of 3.38 ± 0.22 g, 60.04%, 3.30 ± 0.20 g, 58.61%, and 3.20 ± 0.21 g, 56.84%, respectively), without significance (*p* > 0.05) in the different consumption levels between these four gastropods. While the feeding capacity of *P. analis* for the earthworms was the lowest among the foods, the feeding amount was 2.01 ± 0.19 g (35.70%), which was significantly (*p* < 0.05) more than that of the other seven foods with zero consumption, including insects, pork and crucian carp fish meat, freshwater shrimp, and coconut.

## 4. Discussion

The results from this study show that firefly larvae preferred snails within a similar habitat type, highlighting the effect of habitat on food choice. However, further comparison showed obvious differences in the feeding range of the two aquatic lineages (12 vs. 6), suggesting that the feeding habits of aquatic fireflies are not only determined by their freshwater habitats. Differences in their overall dietary preference may result from interspecific diversification of larval morphology and behavior, as is reflected in the developmental pattern of the respiratory organs on their body surface (e.g., respiratory gills of *A. leii* vs. spiracle appearance of *S. substriata*), their predatory behavior (ferocious vs. relatively mild), and their swimming strokes (e.g., only underwater crawling of *A. leii* and eight different stroke patterns of *S. substriata*) [8]. This diversification may have resulted in ecological diversification into freshwater microhabitats with extremely limited dispersal potential for aquatic insects due to ubiquitous fragmentation and the low connectivity of freshwater environments. Similarly, both aquatic Trichoptera and Diptera exhibit exceptional diversity in larval feeding types [20], and the current study extended this finding to beetles.

Studies on the feeding habits of semi-aquatic fireflies and insects in general are extremely limited. Here, we found that both species of semi-aquatic fireflies presented exceptional diversity in larval feeding types, even including coconut, and exhibited omnivory, which is relatively rare at a developmental stage in insects. This finding may help improve beetle life history and reproductive success due to the critical importance of plant-derived resources [21]. We also recorded terrestrial *P. analis* preferably feeding on land snails, which has also been reported in other terrestrial fireflies, including *P. pectoralis* [12] and *Pyrocoelia oshimana* [22]. Moreover, a global survey of experts found that terrestrial fireflies will predate on land snails, earthworms, and other soft-bodied prey [23], as was confirmed in the current study. This suggests that terrestrial gastropods, particularly land snails, are the favorite foods of terrestrial firefly *Pyrocoelia* and that this genus, as strict carnivores, possesses a lower dietary diversity than *A. leii* and semi-aquatic fireflies but higher than *S. substriata* fireflies.

To our knowledge, *A. leii*, *S. substriata*, and *P. qingyu* are the most widely artificially reared aquatic and semi-aquatic firefly species, at least in China. They have been listed as a protected species since June 2023 (http://www.forestry.gov.cn/c/www/gsgg/509640.jhtml (accessed on 5 December 2024), as announced (No. 17 of 2023) by the National Forestry and Grassland Administration and National Park Administration of China). We found a high feeding preference for *C. chinensis*, a commercialized species of freshwater snails, from these two species of aquatic fireflies, and it is the first study to reveal *C. chinensis* as a favorite food of semi-aquatic fireflies. In general, frozen *C. chinensis* can be purchased more easily from the market than fresh and living individuals. Despite this study did not test the feeding habits of fireflies on frozen freshwater snails, previous studies have shown that *A. leii* feeding exclusively on frozen muscle of freshwater snails (*B. purificata*) grew well, with high pupation rate and adult emergence rate [9]. This evidence indicated that readily available muscle products of *C. chinensis* can serve as wild snail alternatives in the artificial feeding of aquatic and semi-aquatic fireflies. Furthermore, previous studies have found a higher nutritional value in pork and freshwater fish meats (i.e., the concentrations of amino acids) than in freshwater snails [9]. However, the larvae of *A. leii* exclusively fed on fish and pork meat for a long time did not rear well (developmental time approximately 210 days), with lower pupation and emergence rates [9]. Therefore, composite artificial diets containing a high proportion of snails and an appropriate addition of pork or fish meat may be better for the promotion of the growth of aquatic and semi-aquatic fireflies. In addition, this study experimentally found that *P. analis* also preferred to feed on *C. chinensis*, second only to the *Bradybaena* genus as wild land snails, showing that *C. chinensis* is also considered a regular food of *Pyrocoelia* fireflies in artificial feeding.

## 5. Conclusions

This study surveyed the larval feeding habits of firefly species across aquatic, semi-aquatic, and terrestrial lineages and performed interspecific and intraspecific comparisons of their preferred foods. These showed obvious differences among and within (e.g., aquatic lineages) various habitat types, but results need to be further verified by a larger study covering more firefly lineages and tested food types (e.g., viscus of domestic animals). Furthermore, analysis of feeding preference showed that the muscle of *C. chinensis*, an easily accessible meat product, may be a substitute for natural snails in the artificial rearing of fireflies, as tested in this study. Certainly, further assessment of the development of firefly feeding on *C. chinensis* is necessary to confirm that the larvae grow and mature well into healthy adults.

## Data Availability

Data are contained within the article.

## Supplementary Materials

The following supporting information can be downloaded at https://www.mdpi.com/article/10.3390/insects15121004/s1. Figure S1. The food preference testing experiment for larvae from *Aquatica leii*, *Sclerotia substriata*, *Pygoluciola qingyu*, *Pygoluciola* sp., and *Pyrocoelia analis*. The 14 foods were sequentially put on the bottom of the boxes (44 cm × 33 cm × 16.7 cm) in circle shape, from 1 to 14, *Sinotaia quadrata*, *Margarya melanioides*, *Cipangopaludina chinensis*, *Macrobranchium nipponense*, pork (*Sus*) meat, fish (*Carassius auratus*) meat, *Tenebrio molitor*, *Aedes aegypti*, *Pheidole megacephala*, *Bradybaena ravida*, *Bradybaena similaris*, *Agriolimax agrestis*, coconut (*Cocos nucifera*) flesh, and earthworm (*Eisenia foetida*). Subsequently, the 100 4th instar larvae after starvation for 24 h were released in the central circle marked by black arrow. Figure S2. Consumption weight of the 100 4th instar larvae of *Aquatica leii* (a), *Sclerotia substriata* (b), *Pygoluciola qingyu* (c), *Pygoluciola* sp. (d), and *Pyrocoelia analis* (e) against *Sinotaia quadrata*, *Margarya melanioides*, *Cipangopaludina chinensis*, *Bradybaena ravida*, *Bradybaena similaris*, *Agriolimax agrestis*, *Macrobranchium nipponense*, *Aedes aegypti*, *Pheidole megacephala*, *Tenebrio molitor*, pork (*Sus*) meat, fish (*Carassius auratus*) meat, earthworm (*Eisenia foetida*), and coconut (*Cocos nucifera*) flesh in 24 h. Table S1. Information of foods tested in this study. Table S2. The weight of the absorbed water of foods for 24 h in aquatic and semi-aquatic environments.
