# Supplementary material for: Larval Feeding Habits of Five Firefly Species Across Aquatic, Semi-Aquatic, and Terrestrial Lineages
Source: Insects. 2024 Dec 18;15(12):1004. doi: 10.3390/insects15121004 (PMC11679764; doi:10.3390/insects15121004)
Supplement: Supplementary file 1 [file insects-15-01004-s001.zip › insects-3332437-supplementary.pdf]

Supplementary Information

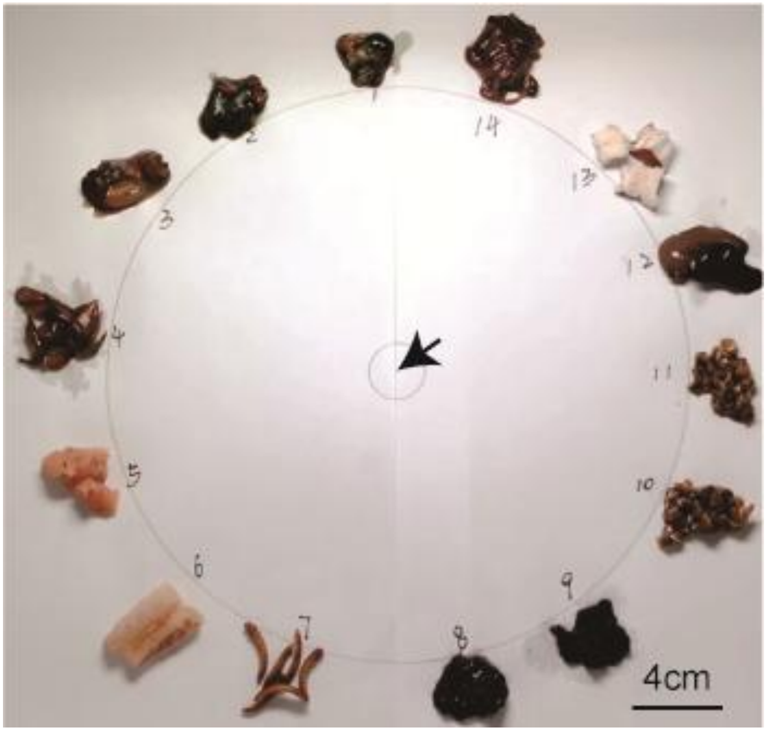

Figure S1. The food preference testing experiment for larvae from *Aquatica leii*, *Sclerotia substriata*, *Pygoluciola qingyu*, *Pygoluciola* sp., and *Pyrocoelia analis*. The 14 foods were sequentially put on the bottom of the boxes (44 cm × 33 cm × 16.7 cm) in circle shape, from 1 to 14, *Sinotaia quadrata*, *Margarya melanioides*, *Cipangopaludina chinensis*, *Macrobranchium nipponense*, pork (*Sus*) meat, fish (*Carassius auratus*) meat, *Tenebrio molitor*, *Aedes aegypti*, *Pheidole megacephala*, *Bradybaena ravida*, *Bradybaena similaris*, *Agriolimax agrestis*, coconut (*Cocos nucifera*) flesh, and earthworm (*Eisenia foetida*). Subsequently, the 100 4th instar larvae after starvation for 24 h were released in the central circle marked by black arrow.

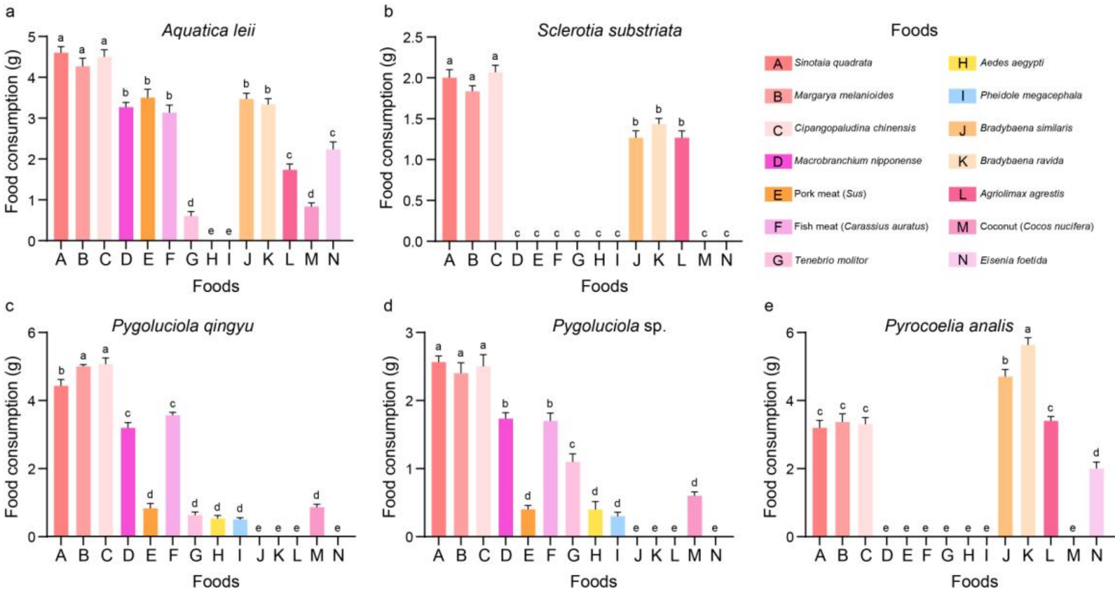

Figure S2. Consumption weight of the 100 4th instar larvae of *Aquatica leii* (a), *Sclerotia substriata* (b), *Pygoluciola qingyu* (c), *Pygoluciola* sp. (d), and *Pyrocoelia analis* (e) against *Sinotaia quadrata*, *Margarya melanioides*, *Cipangopaludina chinensis*, *Bradybaena ravida*, *Bradybaena similaris*, *Agriolimax agrestis*, *Macrobranchium nipponense*, *Aedes aegypti*, *Pheidole megacephala*, *Tenebrio molitor*, pork (*Sus*) meat, fish (*Carassius auratus*) meat, earthworm (*Eisenia foetida*), and coconut (*Cocos nucifera*) flesh in 24 h. Error bars represent S.E. of three biological replications. Different letters above the data columns indicate significant differences at the 0.05 level.

Table S1. Information of foods tested in this study. All these foods are killed and fresh.

Taxon	Names	Types (developmental stages)	Market Price (CNY/kg)#	Coexistence*	The reasons for choosing foods
Freshwater snails	*Sinotaia quadrata*<br>*Margarya melanioides*	Muscle without shell (adult)	Wild collection	AL, SS, PQ, PS	*S. quadrata* and *M. melanioides* as candidate foods in previous studies.
	Cipangopaludina chinensis		25-50		*C. chinensis* is easily accessible, low cost, and environmental-friendly. It was detected to be fed by fireflies.
Freshwater shrimp	*Macrobranchium nipponense*		25-30		Shrimp meat is easily accessible, low cost, with high protein content, and it was detected to be fed by fireflies.
Vertebrates	Pork meat (*Sus*)	Muscle (adult)	40-60	None	Pork and freshwater fish meat is easily accessible, low cost, with high protein content, and as candidate foods in previous studies.
	Fish meat (*Carassius auratus*)		20-30		
Insects	*Tenebrio molitor*<br>*Aedes aegypti*<br>*Pheidole megacephala*	The whole body (adult)	30-40<br>200-300	PA	Three lineages of insects were detected to be fed by fireflies.
Terrestrial gastropod	*Bradybaena similaris*<br>*Bradybaena ravida*<br>*Agriolimax agrestis*	Muscle without shell (adult)	Wild collection		Land snails and slugs as candidate food in previous studies.
Plant fruits	Coconut (*Cocos nucifera*)	White flesh	40-50	None	Coconut flesh was detected to be fed by fireflies.
Annelida	*Eisenia foetida*	The whole body (adult)	15-20	PA	Earthworm was detected to be fed by fireflies.

# If foods are purchasable in the market place in China, their general price ranges were presented.

* AL: *Aquatica leii*; SS: *Sclerotia substriata*; PQ: *Pygoluciola qingyu*; PS: and *Pygoluciola* sp.; PA: *Pyrocoelia analis.*

Table S2. The weight of the absorbed water of foods for 24 h in aquatic and semi-aquatic environments.

Foods	The weight of the absorbed water of foods for 24 h in aquatic environments (g)	The weight of the absorbed water of foods for 24 h in semi-aquatic environments (g)
Sinotaia quadrata	7.81±0.13	6.37±0.16
Margarya melanioides	7.65±0.11	6.21±0.15
Cipangopaludina chinensis	7.72±0.20	6.32±0.17
Macrobranchium nipponense	7.21±0.22	6.24±0.13
Pork meat (*Sus*)	8.26±0.19	6.71±0.09
fish meat (*Carassius auratus*)	7.44±0.24	6.34±0.10
Tenebrio molitor	8.83±0.25	6.96±0.16
Aedes aegypti	8.72±0.24	7.01±0.09
Pheidole megacephala	8.91±0.27	7.11±0.16
Bradybaena ravida	8.42±0.21	6.76±0.12
Bradybaena similaris	8.55±0.18	6.82±0.11
Agriolimax agrestis	8.21±0.18	6.63±0.14
Coconut (*Cocos nucifera*)	7.63±0.16	6.25±0.15
Eisenia foetida	8.37±0.23	6.57±0.11

*Initial weight for each food is 6 g.
